# Case report: Cutaneous metastases as a first manifestation from breast cancer with concurrent gastric metastases

**DOI:** 10.3389/fphar.2024.1356167

**Published:** 2024-03-04

**Authors:** Lulu Xu, Congcong Wang, Xiaoling Yang, Liangliang Dong

**Affiliations:** ^1^ Departments of Oncology, The Affiliated Yantai Yuhuangding Hospital of Qingdao University, Yantai, Shandong, China; ^2^ Department of Obstetrics, Feicheng People’s Hospital, Feicheng, China

**Keywords:** cutaneous metastases, breast cancer, gastric metastases, invasive lobular carcinoma, endocrine therapy

## Abstract

**Background:** Breast cancer represents a leading cause of malignancy among Chinese women, posing a significant health burden. The diagnosis of metastatic breast cancer, particularly to uncommon sites like the skin and stomach, presents distinct challenges.

**Case introduction:** This case report describes a 71-year-old Chinese women with a persistent back rash lasting more than 6 months. Physical examination revealed red papules on her back. Immunohistochemistry confirmed positive for cytokeratin 7(CK7), GATA-3 and GCDFP15, as well as negative staining of cytokeratin 20 (CK20), suggesting breast cancer metastasis. Further evaluation revealed a breast nodule and axillary lymph node enlargement, with biopsies confirming invasive lobular carcinoma (ILC). Abdominal computed tomography (CT) revealed thickening of the gastric and ascending colon walls. Gastroscopy revealed chronic superficial atrophic gastritis. However, gastric metastasis was further confirmed by pathology. The patient initiated endocrine therapy with fulvestrant and exemestane, resulting in rash resolution and stable breast and stomach lesions after 3 months. Overall, the patient is experiencing an improvement in her condition and remains stable while continuing treatment.

**Conclusion:** This case highlights the importance of considering atypical metastatic patterns in breast cancer and the potential efficacy of endocrine therapies in managing such cases. Moreover, it emphasizes the need for vigilance in breast cancer patients, especially those with ILC, as gastrointestinal symptoms may indicate gastric metastasis (GMs). Ultimately, early detection and appropriate treatment strategies, such as endocrine therapy, can contribute to improved outcomes in these challenging cases.

## Introduction

In recent years, the incidence of breast cancer has ranked first among female malignant tumors in China, accounting for approximately 57.27% of case and posing a serious health threat to Chinese women (Zheng et al., 2023). The integration of surgical intervention, chemotherapy, radiotherapy, endocrine, and targeted therapies has markedly enhanced patient survival rates. Nevertheless, breast cancer continues to be a predominant health issue. The predominant histological variants of invasive breast cancer are invasive ductal carcinoma (IDC) and invasive lobular carcinoma (ILC), with IDC constituting about 80% of breast cancers and ILC comprising roughly 5%–15% (Carcoforo et al., 2012). ILC is distinguished by its origin in the lobular units of the terminal duct (Waldman et al., 2019) and demonstrates distinct metastatic behaviors compared to IDC, complicating early detection efforts.

Despite advancements, the scarcity of efficacious targeted treatments for metastatic breast cancer persists due to diagnostic limitations (Liang et al., 2020). Early-stage diagnosis is crucial, as it significantly influences prognosis: the 5-year survival rate for localized breast cancer is 99%, but only 27% for metastatic breast cancer (Wang et al., 2019). Cutaneous metastases (CMs), manifesting in about 24%–50% of breast cancer instances as the primary tumor spreads to skin and soft tissues, presenting as a “fungal breast mass (Huang et al., 2022)” The lymph nodes, bones, lungs, liver, and brain are the most frequent metastatic destinations (Liang et al., 2020). Unfortunately, due to the early appearance of erythema and rashes on the breast’s skin, these patients are often diagnosed at an advanced stage. Metastasis to less common areas such as the back, upper arms, lower abdomen, and notably rare, the buttocks and perianal region, further complicates the disease management. Data on treating CMs are sparse and show little impact on survival rates.

A notably low incidence of gastric metastasis (GMs) from breast cancer was reported by Borst and Ingold, at just 0.26% (Borst and Ingold, 1993). McLemore et al. also demonstrated a relatively low incidence rate of gastrointestinal (GI) metastases originating from breast cancer, about 0.34% (McLemore et al., 2005). A case reported by Güler et al. highlighted a patient with breast cancer-related GI metastasis presenting with acute abdominal pain due to gastric perforation (Güler et al., 2019).

This report uniquely discusses a case of invasive ILC initially manifesting with CMs and accompanied by GMs. The purpose of this article is to provide comprehensive insights into the characteristics, clinical diagnosis, and treatment of such rare cases, thus contributing to the medical knowledge regarding this atypical metastatic pattern in breast cancer.

## Case presentation

### Chief complaints

A Chinese female, who is 71-year-old, came to our hospital, (The Affiliated Yantai Yuhuangding Hospital of Qingdao University) for treatment due to “More than half a year after the discovery of the back rash” in 14 November 2021.

### History of past illness

The patient described no other discomfort prior to this visit and no history of multiple chest wall nodules.

### Physical examination

**FIGURE 1:**
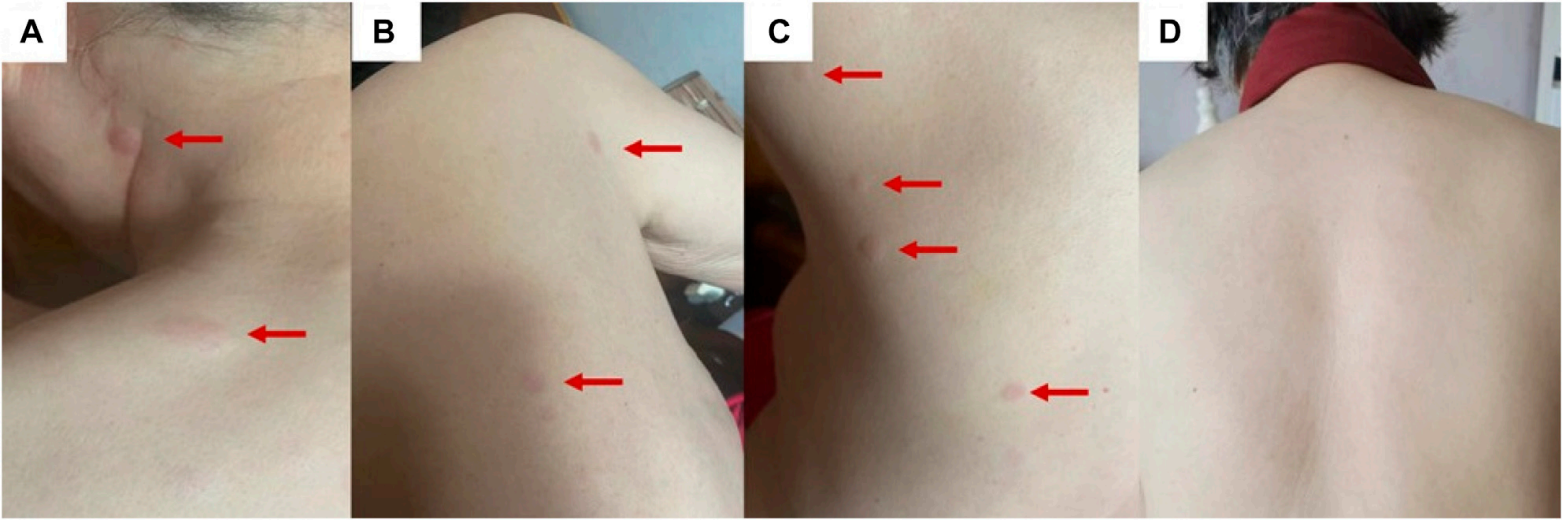
The clinical presentation is multiple red papules scattered on the back **(A–C)**. After 3 months of endocrine therapy, the back rash disappeared **(D)**.

Physical examination revealed multiple scattered red papules on the back, protruding from the surface of the skin with clear boundaries and no itching (Figures 1A–D). No abnormalities were found in the bilateral mammary glands, and no mass was palpated. There is no palpable mass in the abdomen, and there is no tenderness on palpation. There are no positive signs in other parts.

### Preliminary laboratory diagnosis

**FIGURE 2:**
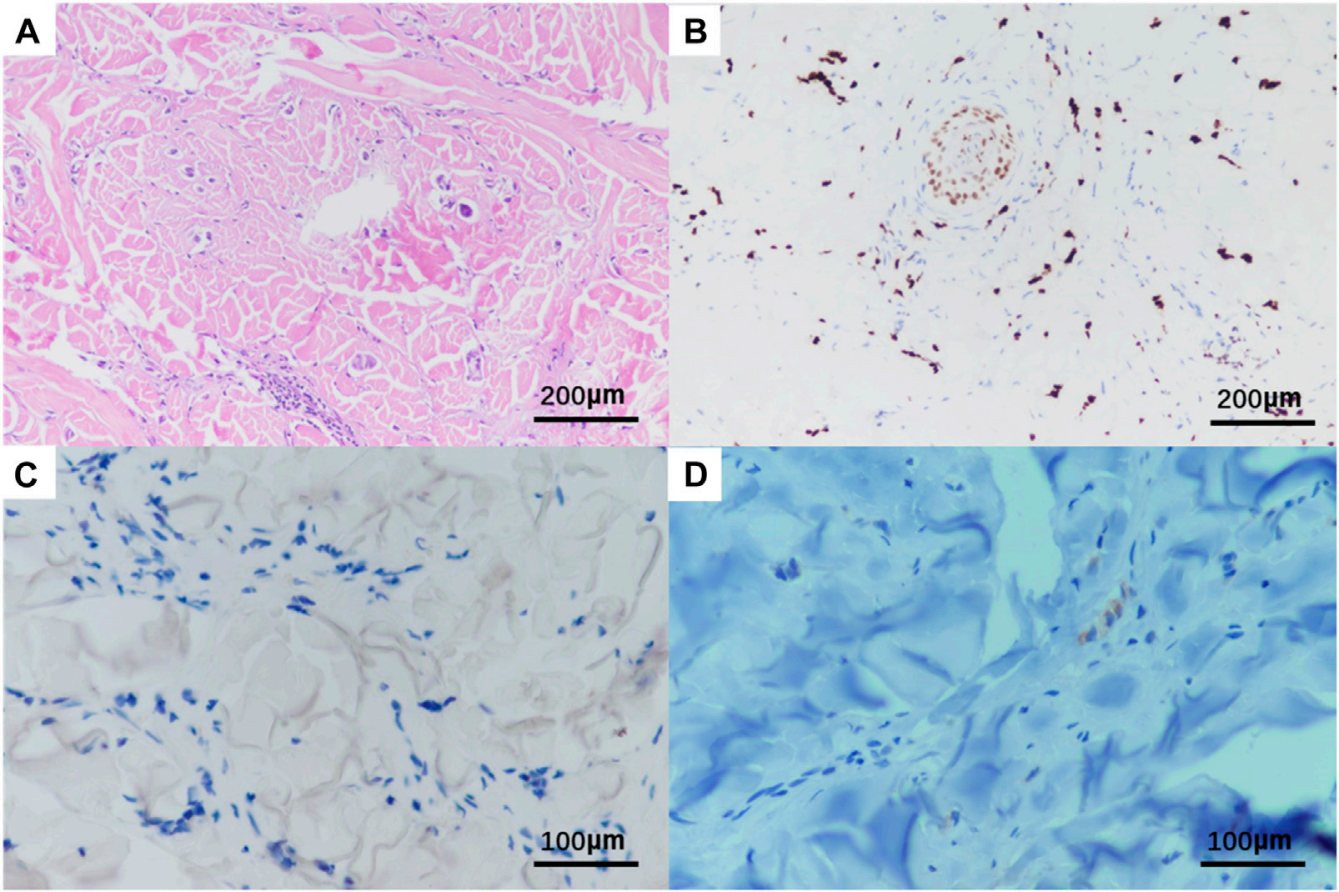
Histopathological HE and immunohistochemistry of patient‘s skin biopsy. Original magnification × 20: **(A)** HE; **(B)** positivity for GATA3; Original magnification × 400: **(C)** negative for E-cadherin; **(D)** positivity for GCDFP15.

A needle biopsy was performed in the dermatology outpatient department of our hospital. Histopathological studies showed mild keratinization in the epidermis of the skin tissue, chronic inflammatory cell infiltration around small vessels in the superficial dermis and subcutaneous adipose septum, moderate and mild heterotypic cells scattered among collagen fibers in the dermis, arranged in a nest-like pattern. Immunohistochemistry showed strong positive for estrogen receptors (ER) and progestogen receptors (PR), both ER and PR percentages are 80%, positive for CK7, GATA-3, GCDFP15 and KI67, as well as negative staining of CK20 and E-cadherin (Figure 2, The image shows only part of the results). Combined with the above results, breast cancer metastasis was considered.

### Comprehensive diagnosis

**FIGURE 3:**
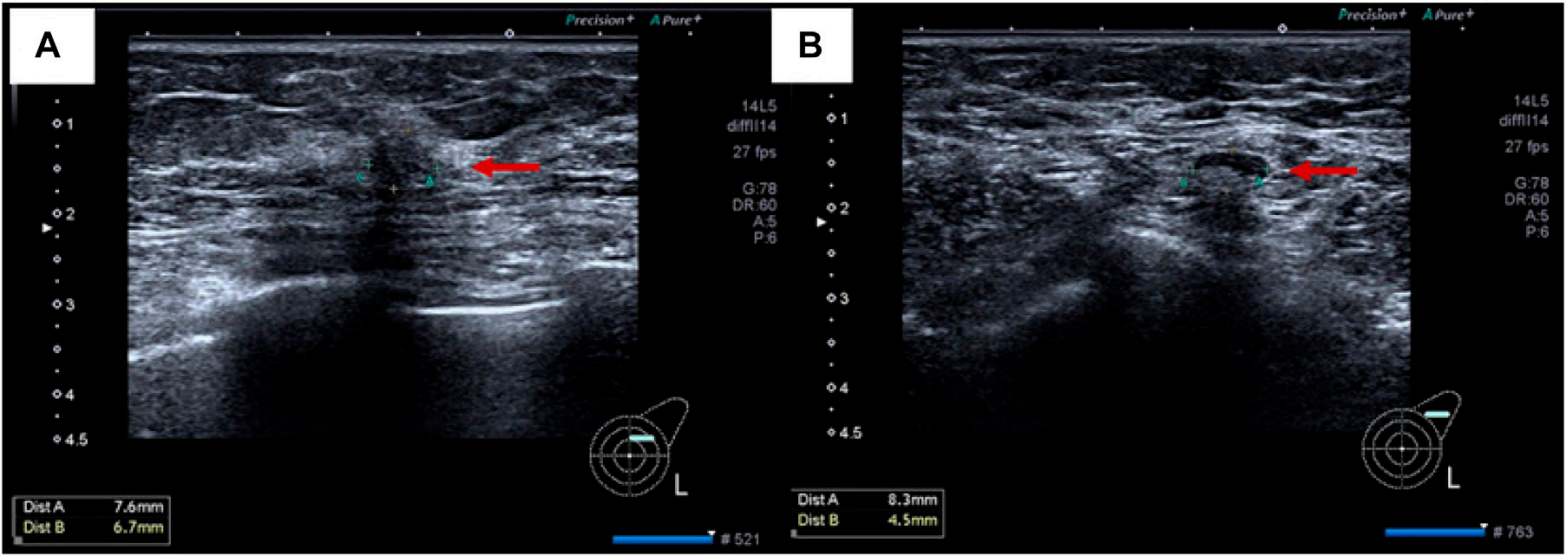
Imaging pictures of the patient at admission. **(A)** breast ultrasound; **(B)** lymph node ultrasound.

**FIGURE 4:**
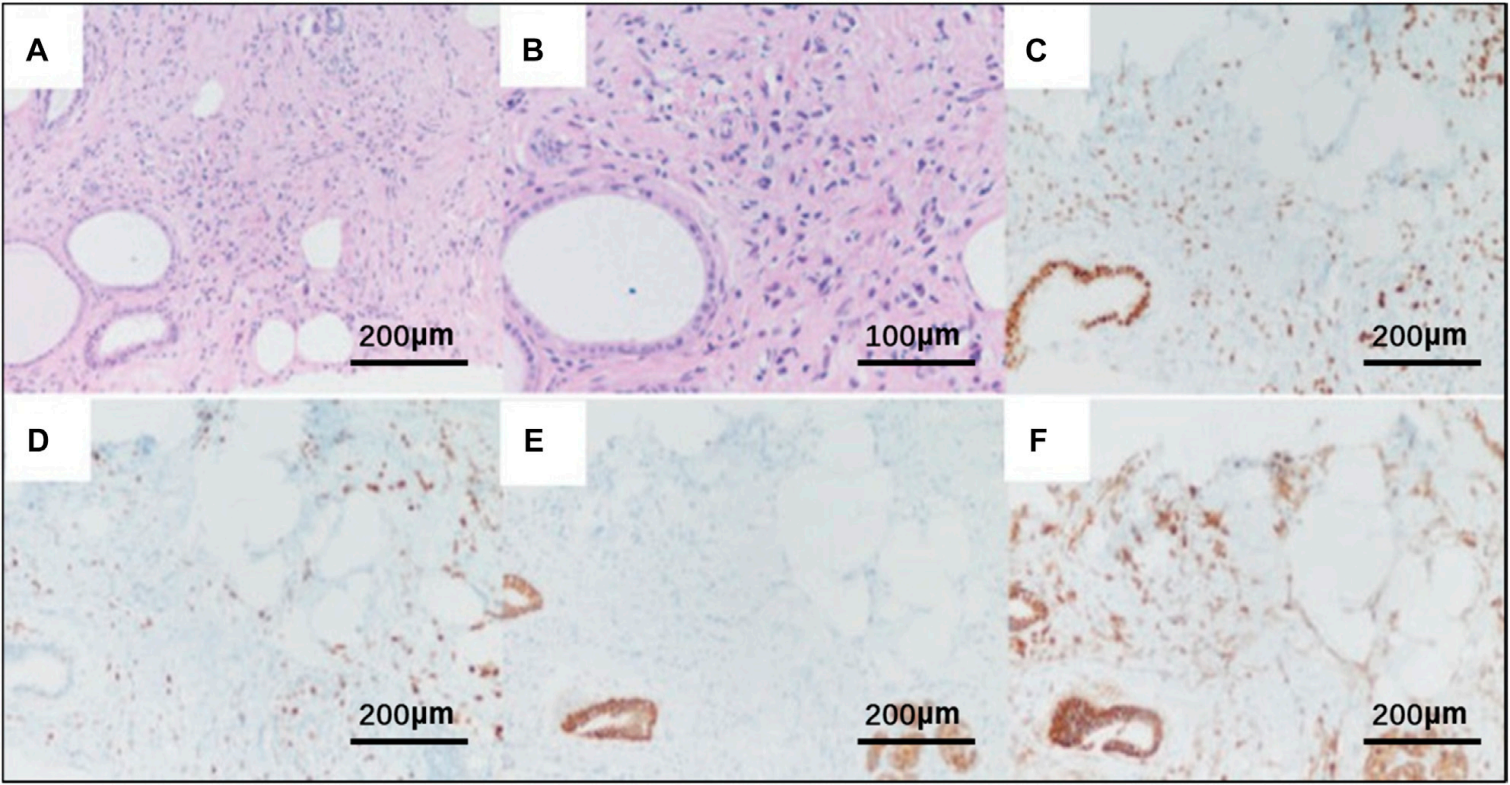
Pathological HE and immunohistochemistry of breast tissue biopsy of the patient. **(A)** HE × 20; **(B)** HE×40; Original magnification × 20: **(C)** strong positivity for the estrogen receptor (ER); **(D)** strong positivity for the progesterone receptor (PR); **(E)** negative for E-cadherin; **(F)** P120 was positive in the cytoplasm.

On 19 November 2021, the patient then underwent a thorough examination. Tumor markers indicate that carbohydrate antigen 153 (CA153) is 126 U/mL (normal range 0–25 U/mL), and no abnormalities are found in the rest. Breast ultrasonography revealed a hypoechoic nodule in the left breast tomography, located 6 cm away from the nipple in the direction of 1–2o’clock, 0.7 × 0.6 cm in size, irregular in shape, aspect ratio<1, unclear boundaries, uneven internal echo, and attenuation of rear echo. There is no obvious blood flow signal within the nodule. Breast imaging reporting and data system (BI-RADS) category 6 (Figure 3A). Enlarged lymph nodes can be seen in the left axilla, the largest being 0.8 cm × 0.4 cm, with full shape, clear boundaries, thickened cortex, clear lymph nodes, and no obvious blood flow signal in lymph nodes (Figure 3B). The pathological findings of the left breast hypoechoic area and left axillary lymph node after biopsy showed that the immunohistochemistry of the left breast hypoechoic area was strong positive for ER (90%) and PR (80%), and negative for human epidermal growth factor receptor 2 (HER2) and E-cadherin, P120 was positive in the cytoplasm, and the positive rate of Ki67 was about 30% (Figure 4). Combined with the morphological and immunohistochemical results, it was consistent with invasive lobular carcinoma (ILC) of the breast.

### Imaging examination

**FIGURE 5:**
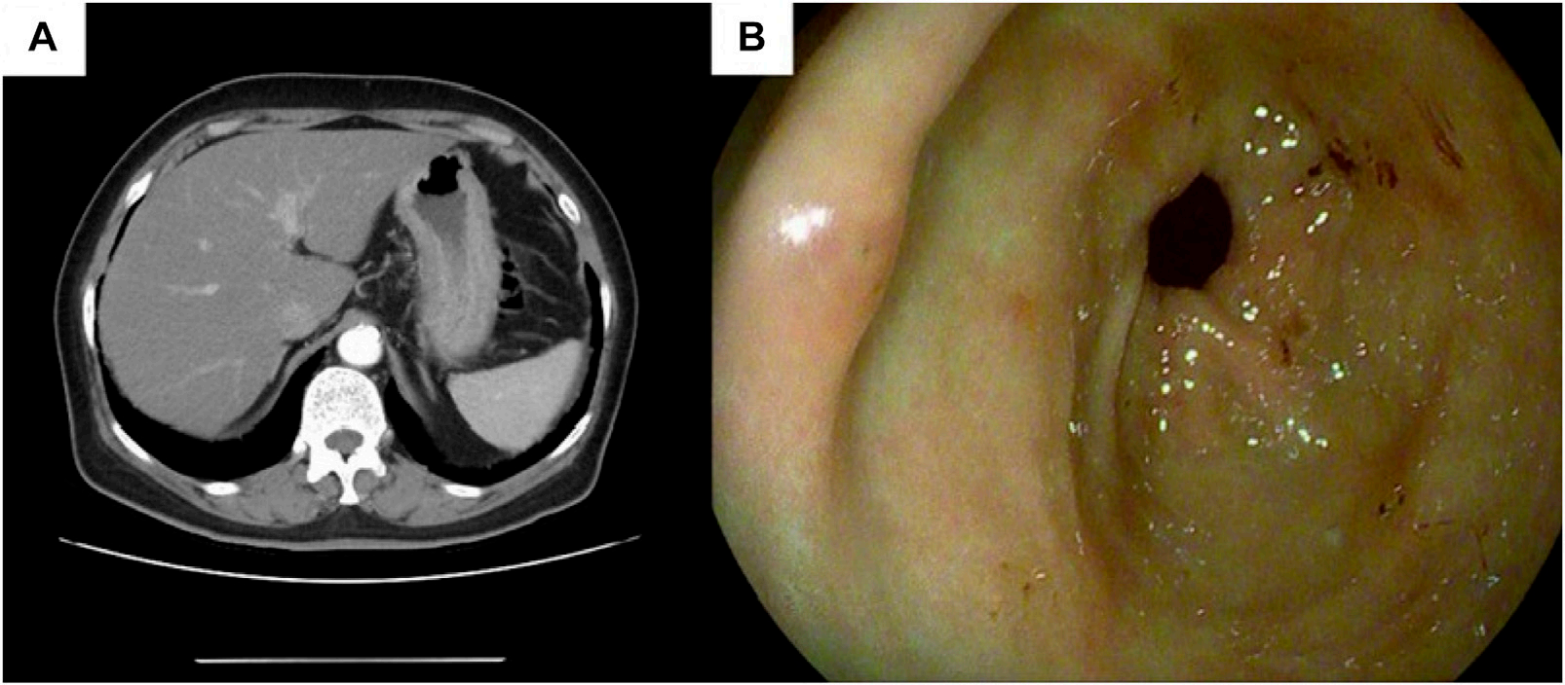
Gastric endoscopy and CT images of the patient. **(A)** Abdominal CT shows gastric wall thickening,the thickest part of which was about 2.3 cm in diameter, involving the whole circumference of the lumen, with a length of about 5 cm; **(B)** Endoscopy prompts chronic superficial atrophic gastritis.

**FIGURE 6:**
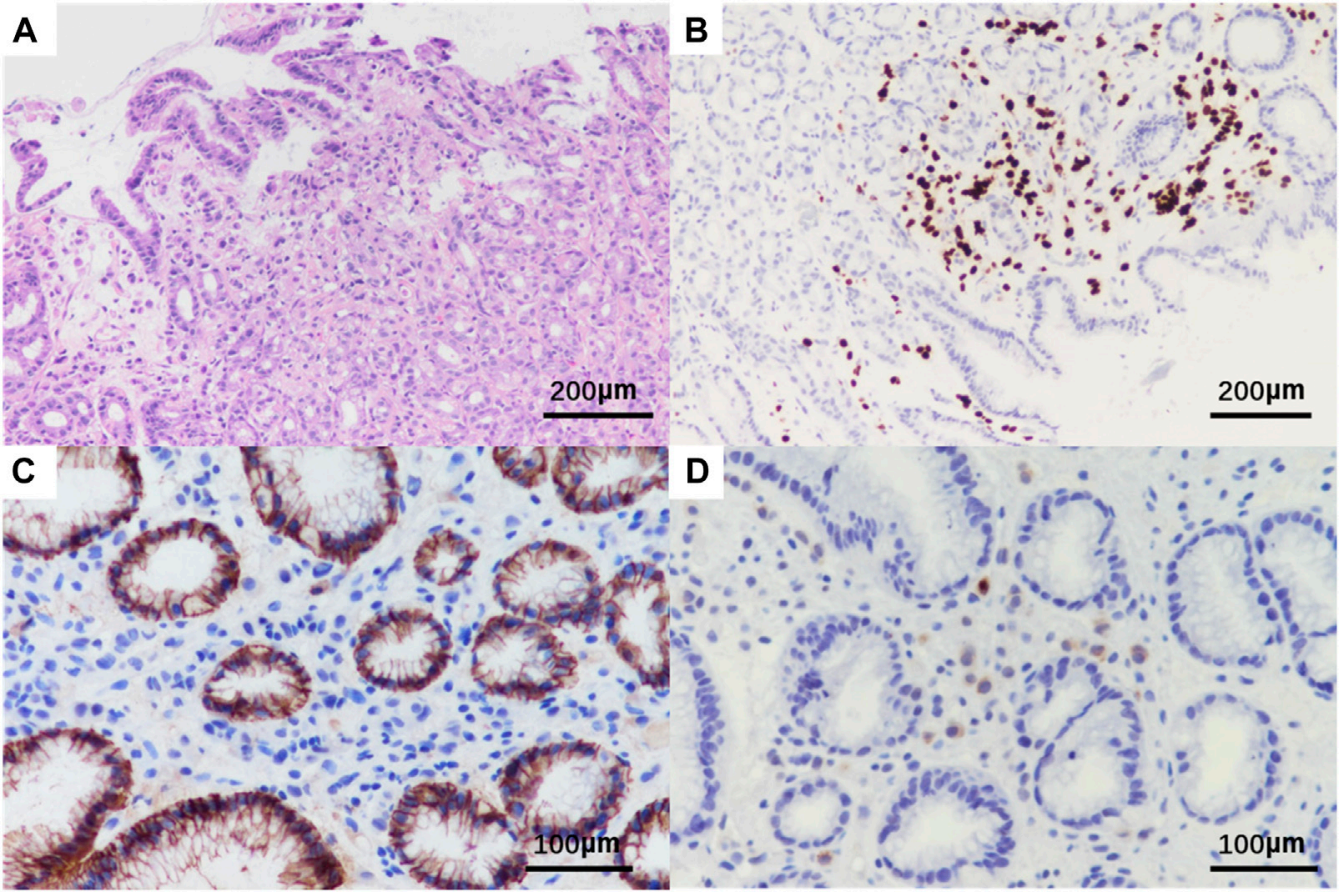
Pathological HE and immunohistochemistry of gastric tissue biopsy of the patient. Original magnification × 20: **(A)** HE; **(B)** positivity for GATA3; Original magnification × 400: **(C)** negative for E-cadherin; **(D)** positivity for GCDFP15.

Subsequent staging tests, including abdominal and chest CT, brain magnetic resonance imaging and, bone scan, revealed synchronous metastases to stomach. The pathological results of left axillary lymph nodes were consistent with the pathological findings of the breast. Abdominal enhanced computed tomography (CT) (Figure 5A) showed poor gastric filling, thickening of gastric wall, and mild to moderate enhancement on enhanced scans. The local intestinal wall of the ascending colon is rough and slightly thick. Gastroscopy and colonoscopy were recommended. Chest CT, brain magnetic resonance imaging and bone scan showed no obvious abnormality. On 21 November 2021, The patient underwent gastroscopy, and under the microscope, congestion and roughness of the gastric antrum mucosa were observed, accompanied by scattered erosive lesions. Two gastric antrum biopsies were performed, with soft texture. Chronic superficial atrophic gastritis is considered (Figure 5B). Pathological examination showed that a small number of heterotypic cells were infiltrated in the lamina propria on the mucosal surface of the antrum. Immunohistochemistry showed strong positive for ER (90%) and PR (80%), positive for CK7, KI67, GCDFP15 and GATA3, and negative for CK20 and E-cadherin, which was consistent with invasive lobular breast carcinoma metastasis (Figure 6, The image shows only part of the results).

### Treatment

Subsequently, endocrine therapy with fulvestrant (Turner et al., 2020) (500 mg, the first two times are 2 weeks apart, and every 4 weeks after that) combined with exemestane [EXE (Turner et al., 2020)] (25 mg, once a day) was started on 14 December 2021. After 3 months of treatment, the patient’s rash disappeared (Figure 1D), and the breast and stomach lesions were stable, and the patient is still receiving treatment.

### Outcome and follow-up

**FIGURE 7:**
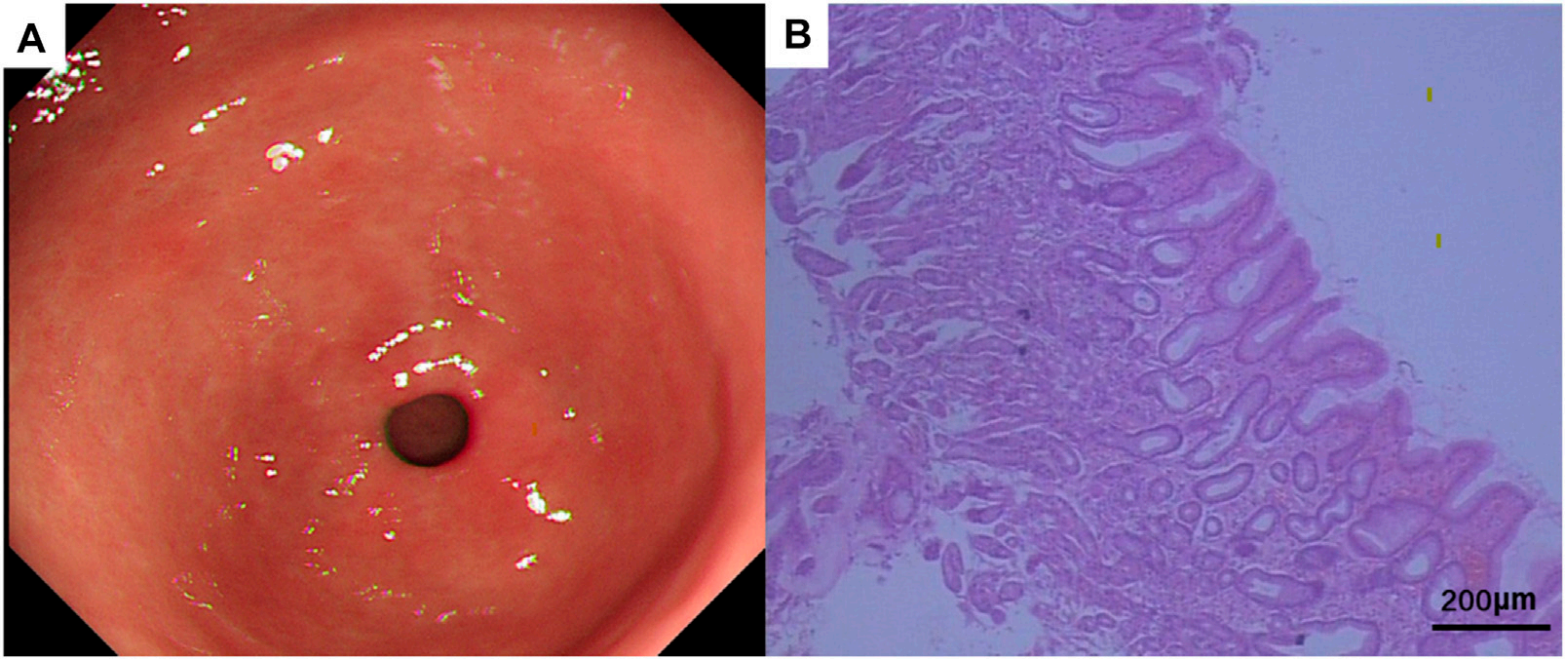
**(A)** Gastroscopic manifestations after treatment. **(B)** Pathological HE of gastric tissue biopsy after treatment. HE × 20.

**FIGURE 8:**
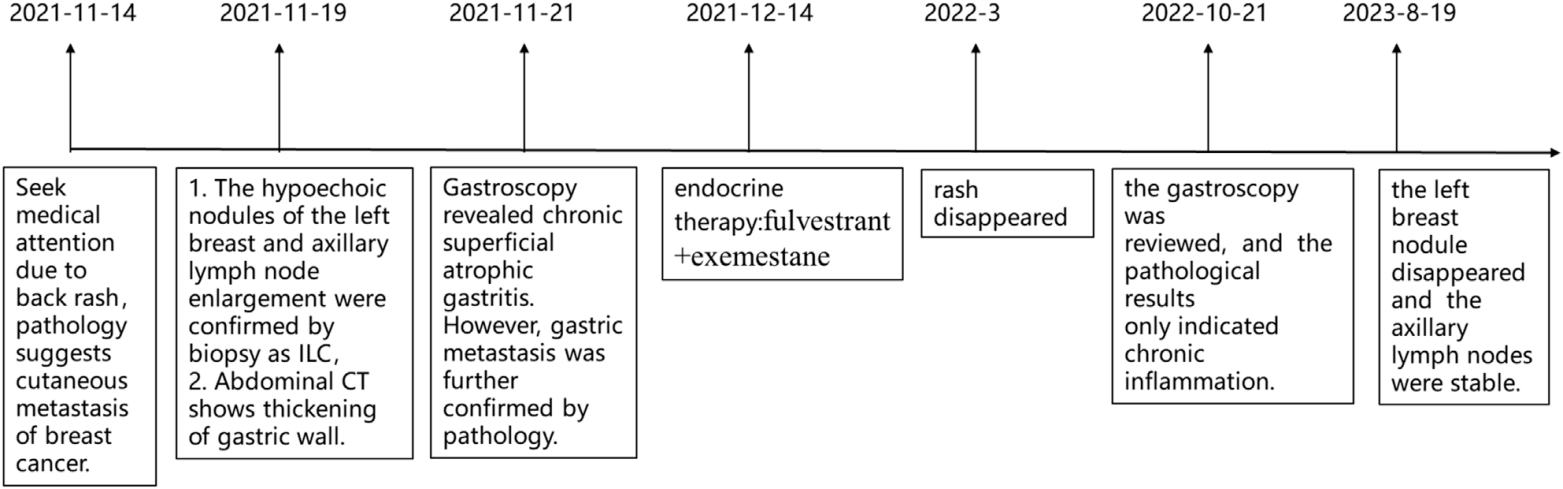
The timeline diagram of patient diagnosis and treatment process.

On 21 October 2022, a follow-up gastroscopy revealed alternating red and white mucosa in the gastric antrum, mainly in red. Scattered congested and rough mucosa can be seen on the anterior and posterior walls of the gastric antrum. Three biopsies were taken at the lesion site of the gastric antrum, with soft texture (Figure 7A). Pathological findings suggest chronic inflammation of mucosal tissue with mild intestinal metaplasia of glandular epithelium (Figure 7B). On 19 August 2023, tumor markers indicated a decrease in CA153 to 46.6 U/mL. Breast ultrasound shows no obvious nodules or liquid dark areas in the left breast layer; Lymph nodes can be seen in the left armpit, with a size of approximately 2 cm × 0.6 cm. The last application of fluvastatin was from 16 September 2023, and EXE continued to be orally administered daily. Our last follow-up was on 16 September 2023. The entire diagnosis and treatment process of the patient is shown in Figure 8.

## Discussion

Research has elucidated that ILC and IDC manifest distinct patterns of distant metastasis (Teo et al., 2018). IDC predominantly metastasizes to bone, lung and liver, while ILC is more prone to gastrointestinal, female reproductive organ, peritoneal, retroperitoneal, adrenal, bone marrow and pleural metastasi (Borst and Ingold, 1993). The unique immunological milieu and specific genomic modifications in ILC are pivotal in its metastatic tendencies. Studies have identified a higher non-synonymous tumor mutation burden in metastatic ILC compared to its primary form, with notable variations in the mutation frequencies of pivotal genes such as CDH1, PIK3CA, TP53, and ERBB2 (Du et al., 2018), and differences in CDH1, PIK3CA, ERBB2, TBX3, NCOR1, and RFWD2 compared to metastatic IDC of no special type (IDC-NST) (Pareja et al., 2020). This indicates that ILC’s metastasis involves distinct genetic alterations compared to IDC. Further research highlights that LumA ILC has a higher proportion of high-immune phenotypes and expresses critical immune checkpoint genes more highly than LumA IDC, indicating possible molecular mechanisms for the different metastasis tendencies between ILC and IDC (Pareja et al., 2020).

ILC is considered a major risk factor for CMs (González-Martínez et al., 2022). Silvia et al. discovered that Luminal HER2-type was more common in patients with CMs, of whom 43.7% were ILC. According to Table 1, we summarized some key factors of ILC triggering CMs, and carried out targeted analysis. About 65.4%–97% of gastric metastases were derived from ILC (Almubarak et al., 2011; Mantiero et al., 2018). At present, the molecular mechanism of metastasis is still unclear. E-cadherin is thought to be associated with increased tumor invasion and metastasis in terms of maintenance of intercellular adhesion as well as loss with EMT or gene deletion (Jeanes et al., 2008; Petrova et al., 2016). Removing E-cadherin in IDC cells led to weaker cell-to-cell connections and a shift towards more dispersed cellular aggregates in suspension (Elangovan et al., 2022). Additionally, the absence of E-cadherin correlated with increased independent growth without attachment and greater resistance to cell death when detached, characteristics that are also present in ILC tumors. Since E-cadherin loss and cytoplasmic localization of p120 catenin are unique to ILC, this may be a useful diagnostic tool for differentiating ILC from IDC (Dabbs et al., 2007; Comprehensive molecular portraits of human, 2012; Pramod et al., 2021). It has been proposed that the hallmark genetic loss of CDH1 plays an important role in the metastatic spread of ILC to abnormal anatomical sites, and that mutations in CDH1 lead to inactivation of E-cadherin and loss of adhesion between tumor cells and other epithelial cells, thereby promoting Invasion and metastasis of tumor cells (Zarrilli et al., 2020). Analysis of invasive breast cancer cases in the Cancer Genome Atlas (TCGA) dataset (http://www.cbioportal.org) showed that 66% (107/162) of ILCs carried CDH1 mutations compared to only 3% (22/741) of IDCs (Cerami et al., 2012; Gao et al., 2013; Pramod et al., 2021).

**TABLE 1:** ILC triggers some risk factors of CMs.

Biology of the cancer	Advanced Stage of ILC
	Lymphatic Spread
	Hormone Receptor Status
	Treatment History
	Tumor Characteristics
	Rare Skin Involvement at Diagnosis
Patient’s individual characteristics	Genetic Factors
	Patient’s Overall Health
	Immune System Function

ILC was prone to luminal A subtype, CDH1 mutations and deletion of E-cadherin mRNA expression (Han et al., 2022). The E-cadherin gene function prevents tumor invasion, and in ILC the deletion of E-cadherin results in an increased capacity for tissue invasion (Vleminckx et al., 1991). Eradicating E-cadherin led to a heightened vulnerability to inhibitors targeting the IGF1R/PI3K/Akt and MEK pathways following the removal of the CDH1 gene (Elangovan et al., 2022). FOXA1 and ER are co-expressed at high levels in endocrine-resistant metastatic breast cancer (Ross-Innes et al., 2012). A higher rate of FOXA1 mutations (7% vs. 2%) and a lower rate of GATA3 mutations (5% vs. 20%) were observed in ILC compared to IDC (Ciriello et al., 2015). Suggesting that ILC and IDC may rely on different mechanisms to regulate ER-mediated transcription (Pramod et al., 2021). A comprehensive study shows that profiles 817 breast tumors, including 127 ILC and 490 IDC cases, providing insights into the molecular differences between ILC and IDC (Ciriello et al., 2015). The study identifies E-cadherin loss, mutations in PTEN, TBX3, and FOXA1 as features enriched in ILC (Ciriello et al., 2015; Teo et al., 2018). It highlights PTEN loss associated with increased AKT phosphorylation, which is highest in ILC, suggesting a unique pathway of tumor progression and potential therapeutic targets.

This patient is part of a unique cohort where CMs were identified before the detection of the primary tumor, initially manifesting as papular nodular lesions on the upper back. Firstly, CMs can serve as the inaugural indication of clinically silent tumors (Virmani et al., 2011), albeit rarely presenting as the initial symptom. Their emergence is potentially linked to the individual’s autoimmune or genetic predispositions (Table 1). Secondly, CMs are postulated to arise through lymphatic or vascular dissemination (Table 1), often manifesting as a recurrence of the primary malignancy (Moore, 2002). Histological types encompass glandular, Indian file, lymphoid embolism of malignant cells between collagen fibers, as well as fibrotic and epidermoid phenotypes (Panasiti et al., 2009; Waldman et al., 2019). The incidence of cutaneous metastasis in breast cancer is about 23.9% (Lookingbill et al., 1993). But compared with other solid malignancies, breast cancer has the highest incidence of skin metastases, accounting for about 70% (Khodair et al., 2021). They typically occur months or years after breast cancer diagnosis and often coincide with visceral metastases (Hu et al., 2008) It has been reported that the occurrence of CMs is associated with poor prognosis (González-Martínez et al., 2022). Usually, the prognosis depends on the type (Table 1) and behavior of the primary tumor, and the expected survival at diagnosis is less than 1 year (Cox and Cruz, 1994). CMs present with nonspecific clinical manifestations, complicating the differentiation from other benign conditions.

CMs of breast cancer are most common in the chest, followed by the head and neck, back, and abdomen. The most prevalent clinical manifestation is solitary erythematous invasive papules and nodules (80%) (Prabhu et al., 2009), usually ranging from 1–3 cm in diameter (Busbait et al., 2022). With disease progression, these nodules may ulcerate or undergo infection. Commonly, patients with CMs at mastectomy scars are erroneously diagnosed with surgical site infections (Waldman et al., 2019). Reports have indicated that skin metastases can spread during core needle biopsies, underlining that the notable incidence of intra-scar metastases also mirrors tumor dissemination during surgical interventions. Consequently, securing a biopsy sample is imperative for affirming the diagnosis in individuals suspected of having breast cancer skin metastases, as histological evaluation of the biopsy may reveal cellular proliferation akin to the primary neoplasm. Breast cancer metastasis diagnosis can be validated by detecting markers like cathepsin D among other antigens (Waldman et al., 2019). The sensitivity of certain diagnostic imaging modalities is low, with mammography and ultrasound showing sensitivity rates of 57%–81% and 68%–98%, respectively. Therefore, combining these with highly sensitive MRI (93%) may lead to a successful diagnosis (Busbait et al., 2022). Moreover, individuals with a history of breast cancer ought to undergo comprehensive skin examinations, even years subsequent to breast cancer surgery and chemotherapy. Conversely, the persistence of an extended rash should warrant further evaluation for early detection of breast cancer.

We present an exceedingly rare instance where breast cancer and GMs were identified subsequent to the initial detection of CMs, despite the primary tumor being diminutive. The stomach is an uncommon site of tumor metastasis, with a reported incidence of 0.2%–0.7% according to clinical and autopsy results (De Palma et al., 2006; Namikawa and Hanazaki, 2014). Primary malignancies most frequently metastasized to the stomach included breast cancer (27.9%), lung cancer (23.8%), esophageal cancer (19.1%), renal cell carcinoma (7.6%), malignant melanoma (7.0%) (Namikawa and Hanazaki, 2014). Although GMs from breast malignancies are the most common, the incidence is only 0.3% in clinical reports and 2%–18% in autopsy case reports (Taal et al., 1992; Taal et al., 2000). The average time from breast cancer diagnosis to detection of GMs is estimated to be 5–8 years (De Palma et al., 2006; Almubarak et al., 2011; Xu et al., 2017). At the time of diagnosis of GMs, 90%–94% of patients had concurrent metastases (Cormier et al., 1980; Borst and Ingold, 1993), there are only a few case reports of GMs diagnosed before or at the same time as breast cancer.

GMs of breast cancer and primary gastric adenocarcinoma are very similar in clinical symptoms, imaging, gastroscopic findings and pathological morphology, and are often difficult to distinguish. Predominantly, patients with GMs experience non-specific gastrointestinal symptoms like anorexia, epigastric pain, indigestion, nausea, fever, and weight loss (Tang et al., 2020), with acute abdominal pain due to gastric perforation being a rarity (Koike et al., 2014). The condition usually entails diffuse involvement of the gastric wall, characterized by linitis plastica and predominantly affecting the submucosal and seromuscular layers (Tang et al., 2020). Some authors have suggested that linitis plastica is the most common type of gastric metastasis in breast cancer (73%–83%) (Taal et al., 1992; Eo, 2008). It resembles Borrmann type 4 advanced gastric cancer with diffuse hypertrophy and sclerosis of the gastric mucosal folds and deep invasion of the submucosa and muscularis propria (Geada et al., 2020). Given that GMs predominantly reside in the submucosa and muscularis propria, sparing the superficial mucosal layer, there is a high likelihood of endoscopy yielding false-negative results. Therefore, if the index of suspicion is high, unconventional techniques such as macroscopic biopsy or endoscopic ultrasound-guided fine-needle aspiration cytology should be used whenever possible (Geada et al., 2020). This patient had no symptoms of gastric discomfort, and abdominal CT revealed thickening of the stomach wall, so a gastroscopy was performed. Nonetheless, the endoscopic examination only revealed chronic superficial atrophic gastritis, with no malignancy detected, underscoring the critical role of endoscopic biopsy in such cases.

In cases lacking pertinent clinical history, pinpointing the origin of metastatic cancer can prove challenging. However, metastases often exhibit similar histopathological features as the primary tumor. Diagnosis is relatively easy due to the convenient sampling of skin lesions. However, cancer cells metastasized to the stomach predominantly present as poorly differentiated adenocarcinoma and signet-ring cell carcinoma, complicating their differentiation from primary gastric adenocarcinoma on conventional HE stained sections. The distinction between primary and metastatic must rely on immunohistochemical examination. In this case, the primary tumor, skin and GMs were all strongly positive for ER and PR. Nevertheless, the ER positivity rate in female gastric cancer patients is between 26.6% and 31%, and for PR, it is between 11.9% and 20.6% (Karat et al., 1999). Moreover, even if the primary breast tumor is ER and PR positive, GMs might still test negative for these receptors (Kim et al., 2018), indicating that ER and PR status alone cannot conclusively determine tumor origin. Since HER2 has a comparable positive rate (less than 5%) in ILC and low-adhesion gastric cancers, it is also not an appropriate diagnostic marker (Mantiero et al., 2018). The combination of three antibodies, GCDFP15, mammaglobin and GATA-3, is often used to identify breast cancer metastasis. The sensitivity and specificity of the three expressions in breast cancer were 5%–74% and 9%–100%, 7%–84% and 85%–100%, 32%–100% and 7%–93%, respectively (Gown et al., 2016). In addition, breast tissues were mostly CK7(+)/CK20(−) and gastrointestinal tissues were mostly CK7(−)/CK20(+) (Xu et al., 2017). The immunohistochemistry of CMs and GMs in this patient showed CK7(+), CK20(−) and (+). It is suggested that the tumor may originate from the breast. Furthermore, immunohistochemical staining of this patient showed E-cadherin (−) and P120 (cytosolic +), suggesting that the pathological type was lobular breast carcinoma. Combined with the patient’s medical history, cutaneous and gastric metastasis from ILC of the breast could be clearly diagnosed. In summary, by detecting the expression of specific protein markers by immunohistochemistry, doctors can identify whether metastatic lesions are associated with primary breast cancer, or whether they are associated with other types of cancer. In particular, for patients with no history of breast cancer as in this case, it is helpful to formulate accurate diagnosis and treatment plan, and improve the accuracy of patient treatment effect and prognosis assessment.

CMs in conjunction with visceral metastasis signify advanced disease and a grim prognosis, with mortality typically occurring within a year of diagnosis (González-Martínez et al., 2022). Xu et al. found that pre-gastric involvement metastasis to multiple organs was an independent predictor for overall survival reduction. At present, there is no standard guideline for the treatment of CMs and GMs (Rech et al., 2023), and the treatment principles for advanced breast cancer are mostly followed. Systemic therapy, encompassing chemotherapy [including anti-HER2 therapy (Huang et al., 2022)] and endocrine therapy, is the mainstay of treatment. GMs from breast cancer are generally not treated surgically unless acute complications occur (Kim et al., 2018). Anti-HER2 drugs, including chemotherapy drugs specifically used for HER2-positive breast cancer (Huang et al., 2022) such as trastuzumab, ado‐trastuzumab emtansine, and lapatinib, have been employed. In one case, patients with HER2+ skin lesions experienced complete regression after initiating ado-trastuzumab emtansine treatment, with the response lasting up to 135 weeks (Giarratano et al., 2018; Huang et al., 2022). But XU et al. suggested that surgical intervention and chemotherapy did not significantly prolong OS, while endocrine therapy proved to be an effective strategy (Xu et al., 2017). This may be because most patients with GMs are from ILC, and their hormone receptors are usually positive, mainly luminal-type, which is relatively sensitive to endocrine therapy. Endocrine therapy has been reported to play an important role in improving patient survival (Busbait et al., 2022). Over an 8-year follow-up period, tamoxifen showed a disease-free survival (DFS) and OS of 66% and 74%, respectively, compared to 82% and 89% for letrozole (Metzger Filho et al., 2015; Busbait et al., 2022). There is a case report that CMs from ILC is rapidly and completely relieved with aromatase inhibitor (Parietti et al., 2022), especially in ER+ (Huang et al., 2022). Aromatase inhibitors are sometimes used as maintenance therapy after controlling CMs with conventional means, such as chemotherapy, radiation, and surgery. In particular, the skin lesions partially or completely resolved, and they survived within 2–5 years of follow-up. In addition, EXE and everolimus (EVE) have been reported to better control metastatic lesions in the skin (Li et al., 2022). For patients with hormone receptor positive advanced breast cancer, NCCN guidelines recommend aromatase inhibitor + CDK (Cyclin-dependent Kinase) 4/6 inhibitor or fulvestrant + CDK4/6 inhibitor. CDK4/6 inhibitor is more expensive, and the patient’s economic conditions are poor, so he did not choose the drug. One clinical study demonstrated that Ribociclib combined with Fulvestrant had a 67.0% OS for 36 months, which was higher than Fulvestrant alone (Slamon et al., 2020). Ultimately, endocrine therapy with fulvestrant combined with EXE was administered, leading to satisfactory disease control, with ongoing treatment.

This highlights the uniqueness of each case, emphasizing the need for personalized prognosis discussions based on all relevant clinical and pathological factors. In this rare case, a 71-year-old Chinese female initially presented with CMs as her primary symptom, which led to the subsequent diagnosis of ILC of the breast. Further evaluation revealed synchronous GMs from breast cancer, an exceptionally uncommon occurrence. The diagnosis relied heavily on immunohistochemistry, as the clinical presentation and histopathological features resembled primary gastric adenocarcinoma. It underscores the importance of thorough evaluations in patients with atypical metastatic patterns and the pivotal role of immunohistochemistry in confirming tumor origins.

## Conclusion

In conclusion, CMs and GMs of breast cancer represent uncommon clinical phenomena, particularly when CMs precede the identification of the primary tumor. CMs and GMs often signify advanced disease and poor prognosis, emphasizing the urgency of precise diagnosis and timely intervention. In the absence of relevant clinical history, it is difficult to determine the primary site of metastatic cancer. Without a detailed clinical history, pinpointing the origin of metastatic cancer can be challenging. Furthermore, the symptoms associated with these metastatic sites are often non-specific, leading to potential diagnostic oversight and misdiagnosis. In this context, the interaction between the clinician and the pathologist is critical. The appearance of persistent papular nodular skin lesions necessitates immediate pathological biopsy. The discovery of CMs usually coincides with multiple metastases from other organs, warranting a thorough patient evaluation. Particularly for patients with ILC of the breast, gastrointestinal symptoms should prompt urgent endoscopic biopsy, with immunohistochemical staining aiding in lesion characterization. Early diagnosis and optimal treatment can delay disease progression and reduce mortality. Treatment strategies, including endocrine therapy and targeted agents, must be customized based on the tumor’s molecular profile.

## Data Availability

The raw data supporting the conclusion of this article will be made available by the authors, without undue reservation.

## Abbreviations

BI-RADS, breast imaging reporting and data system; CA153, carbohydrate antigen 153; CDX2, caudal-related homeobox 2; CK20, cytokeratin 20; CK5/6, cytokeratin 5/6; CK7, cytokeratin 7; CMs, Cutaneous metastases; CT, computed tomography; ER, estrogen receptors; EVE, everolimus; EXE, exemestane; GMs, gastric metastases; HER2, epidermal growth factor receptor 2; IDC, invasive ductal carcinoma; ILC, invasive lobular carcinoma; OS, overall survival; PR, progestogen receptors; TTF-1, thyroid transcription factor-1.
